# Comparative Evaluation of Explicit Solvent Models for RNA-Ligand Docking

**DOI:** 10.1021/acs.jcim.6c00498

**Published:** 2026-05-21

**Authors:** Laura Almena Rodriguez, Christian Kersten

**Affiliations:** † Institute of Pharmaceutical and Biomedical Sciences, 9182 Johannes Gutenberg-University Mainz, Staudingerweg 5, 55128 Mainz, Germany; ‡ Institute for Quantitative and Computational Biosciences, Johannes Gutenberg-University, BioZentrum I, Hanns-Dieter-Hüsch Weg 15, 55128 Mainz, Germany

## Abstract

The interest in targeting RNA with small molecules is increasing continuously. However, structure-based drug design approaches have been reported rarely so far. Major challenges in RNA-ligand docking include ligand-induced conformational changes, ions and solvation which hamper successful applications in prospective virtual screenings. We examined the influence of explicit solvent inclusion on RNA-ligand docking performance using crystallographic water sites as well as the computational solvation models 3D-RISM, GalaxyWater-CNN and waterdock_fxx in combination with FlexX, FlexX with HYDE rescoring, GOLD and LeadIT docking. The redocking study with 92 RNA-ligand complexes underlined that the benefit of solvent consideration is highly target-specific and resolution-dependent reaching on average accurate pose predictions of around 70% for all structures and only 35% for low-resolution structures for FlexX, GOLD and LeadIT. HYDE performed slightly worse on average with an overall 50% accurate pose prediction and varying impact of predicted solvent. Success rates of structures lacking experimental solvent information were improved by involving predicted water sites. 3D-RISM predictions showed most robust results across all resolutions, improving success rates by up to 30% for low-resolution structures in combination with LeadIT. In addition, NMR and ion-free structures were found to be more challenging in pose prediction accuracy compared to ion-containing X-ray structures. Cross-docking studies across five representative RNA targets demonstrated improvements for hydrated dockings, while different binding site conformations indicated RNA dynamics as an additional challenge. The best cross-docking setup was partially deducible from the corresponding redocking setup revealing great potential to advance virtual screenings by the inclusion of explicit solvent sites.

## Graphical abstract

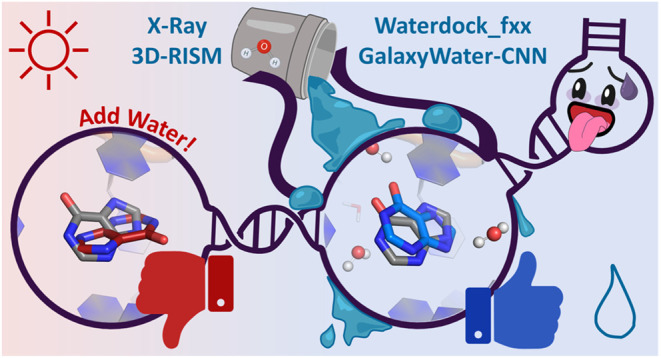

## Introduction

### RNA as a Target for SBDD

RNA plays a central role in biological systems and is essential for both transcription and translation of genes into proteins. In addition, RNA is involved in multiple regulatory processes governing gene expression. Interest in RNA as a potential drug target has increased substantially in recent years. While only approximately 1.5% of the human genome is translated into proteins, an estimated 70–90% is transcribed into RNA.^1,2^ Consequently, targeting RNA rather than proteins broadens the landscape of potential drug targets and holds great potential to circumvent undruggable proteins by addressing their corresponding mRNAs.^3^ Therapeutically relevant RNA targets can be modulated by classical small molecules.^3^ Exploiting the natural metabolite binding site of bacterial riboswitches^4,5^ (RS) or addressing viral RNAs,^6,7^ provides valuable opportunities to develop novel antibiotics and antiviral agents. Moreover, dysregulated human RNAs represent promising drug targets, as highlighted in recent years. Beyond mRNAs and rRNAs, noncoding RNAs (ncRNA) such as microRNAs or long ncRNAs represent a major fraction of the human transcriptome. They are involved in the regulation of gene expression, and their dysregulation is associated with several diseases including cancer.^8,9^ The splicing modifier risdiplam was the first RNA-targeting small molecule approved by the FDA for the treatment of spinal muscular atrophy in 2020.^10,11^ Similar to risdiplam,^10^ many RNA-binding small molecules have been discovered through phenotypic screenings, followed by high-throughput screenings (HTS) or fragment-based approaches.^2,3,12^ Prospective virtual screening (VS) campaigns for RNA targets are rarely reported in literature.^13–18^ Although structure-based RNA-ligand design and computational approaches, such as molecular docking, are gaining increasing attention, they continue to face distinct challenges.^3,19,20^ In comparison to proteins, RNAs exhibit higher conformational flexibility,^21,22^ carry multiple negative charges due to their phosphate backbone—partially shielded by surrounding cations—and typically present binding sites that are more solvent-exposed and shallow.^20^ Conventional docking tools were primarily developed for protein targets, which need to be carefully validated before being transferred to and applied in RNA-targeting VS workflows.^17,23–27^ Several protein-based docking tools have been reported to be, in principle, applicable to RNA ligand docking and VS studies.^17,24,25^ Given that docking performance can vary significantly depending on the target structure and docking tool used,^25,28^ scoring functions as well as RNA-ligand docking algorithms have been adapted and newly developed for nucleic acid^28–31^ docking or for RNA^32–37^ in specific. Although these RNA-focused tools often perform comparable to or better than protein-based docking approaches transferred to RNA,^17,23,26,27,34,35^ their reported results remain difficult to interpret and elusive. This is largely due to the use of different benchmark data sets as well as varying success criteria. One of the major limitations in validating docking tools for RNA-ligand SBDD is the lack of high-resolution crystal structures available in the Protein Data Bank (PDB).^19,38^ Currently, approximately 160,000 protein–ligand complex structures and only around 3000 RNA-ligand X-ray complexes have been published in the PDB.^38,39^ The actual number of suitable RNA-ligand complexes for drug design is even smaller. A curated collection of RNA-ligand complexes determined by X-ray diffraction, solution nuclear magnetic resonance (NMR) spectroscopy and cryo-electron microscopy (EM) was implied in the Harnessing RIBOnucleic acid—Small molecule Structures (HARIBOSS) database in 2022. This database contains 533 RNA-only ligand complexes comprising 456 X-ray, 65 NMR and 12 cryo-EM structures from the PDB.^40,41^ While the number of structures released annually in the PDB has stagnated for X-ray^42^ and has declined for NMR^43^ structures, EM-based^44^ derived structure releases have increased steadily. Cryo-EM has emerged as a promising method for RNA structure determination, as it enables the visualization of different RNA conformations that often hampers X-ray crystallographic analysis.^45,46^

### Solvation

X-ray crystallography represents the primary experimental method for resolving explicit water molecules in target structures. However, even at resolutions better than 2 Å, the assignment of water molecules within electron density maps remains challenging and determination of water orientations is not possible.^47^ Despite these limitations, experimentally derived solvent information provides valuable guidance for SBDD. Water molecules located in ligand-binding pockets are often integrated in interaction networks involving the target, the ligand, or neighboring solvent or ion molecules, which restrict their mobility compared to bulk water.^48–51^ From a thermodynamic perspective, the removal of such ordered water molecules upon ligand binding can improve binding affinity, as the associated gain in entropy may favor complex formation.^48,52–56^ However, this effect is diminished if enthalpic interactions previously formed between the water molecule and the target are not adequately compensated by ligand-target interactions, potentially leading to reduced binding affinity. This indicates that certain water molecules are better regarded as structural components of the binding site rather than as displaceable solvent.^48,53,54,57^ Beyond affinity, structured water molecules can also contribute to ligand selectivity.^58,59^ Nevertheless, a major challenge in molecular docking remains the reliable prediction of solvation sites as well as their displacement or retention upon ligand binding.^53,55^ For RNA targets, this challenge is further amplified by their highly dynamic and solvent-exposed binding environments.^20^ In addition, the thermodynamic properties of individual water molecules are difficult to characterize experimentally or computationally.^53,55^ Solvation plays a crucial role for RNA by mediating structural interactions and enabling its intrinsic conformational flexibility.^60^ Notably, the choice of solvent model can have an even stronger impact on RNA structure than ion concentration as indicated in a molecular dynamics study.^61^

### Hydrated Docking

To compensate for the experimental limitations in resolving structural water molecules, a broad range of computational methods has been established to predict solvation sites, primarily for proteins. These methods can be categorized into interaction-based water site predictions, free energy-based approaches, and knowledge-driven methods, the latter can be coupled to deep-learning approaches.^62,63^ In parallel, a few water site prediction tools have been developed for nucleic acids and have been benchmarked against crystallographic data showing promising results.^64,65^ Despite these advances, the benefit of incorporating experimentally derived or predicted hydration into protein–ligand docking workflows remains ambiguous. Differences in solvent handling features of different docking programs complicate direct comparison across studies. While some investigations reported negligible^66^ or only small^67^ improvements in redocking accuracy, others—ranging from case studies^68–73^ to larger statistical analyses^74–78^ —demonstrated enhanced predictive performance when structural water molecules were considered. Two cross-docking studies suggested that accounting for water molecules can improve cross-docking accuracy.^72,74^ However, these effects are not uniform across targets, as the most suitable pairing of solvent model and docking tool can differ. Notably, consensus strategies that integrate multiple solvent models and docking tools yielded improved performance in both, redocking and cross-docking evaluations.^74^ At the same time, excessive inclusion of water molecules may impose unnecessary spatial constraints on ligand sampling, effectively biasing dockings toward predefined binding modes. Although such constraints can enhance docking performance in redockings, they reduce robustness and may lead to failed cross-dockings and consequently reduced predictive power.^67,70,71,74^ For RNA targets, systematic, statistical evaluations of the impact of explicit solvent on docking performance were not reported, yet. Initial docking studies for specific targets showed improvements when crystallographic water molecules,^25^ selected key water molecules,^13,17^ or predicted solvation sites^79,80^ were included. The observed effects were dependent on the docking tool applied.^25^ One of these investigations focused exclusively on aminoglycosides-RNA complexes.^80^

SBDD and prospective virtual screening approaches remain rare in RNA-ligand design. The intrinsic flexibility, negative charge and solvent-exposed binding sites of RNA require careful validation of docking tools *prior* to their application to RNA targets. The importance of solvation for RNA suggests that consideration of solvation sites during molecular docking may improve predictive performance. The present work examines the influence of explicit solvent inclusion on RNA-ligand docking performance, focusing on the role of crystallographic and computationally predicted solvation sites. Beyond redocking, the impact of water molecules on cross-docking performance was examined across five representative cases. We employed a modified data set from literature^81^ comprising 92 RNA-drug-like-ligand complexes. Four docking setups were used—FlexX,^82^ FlexX with HYDE^83,84^ rescoring, GOLD,^85,86^ and LeadIT^82^—each applied in combination with solvent-free structures, structures retaining crystallographic water molecules, and structures containing solvation sites predicted by 3D-RISM,^87^ GalaxyWater-CNN,^63^ and waterdock_fxx.^74^

## Materials and Methods

### Redocking Data Set

For redockings, we used a subset of the previously published DrugPred_RNA^81^ data set, from which rRNA-ligand complexes, redundant structures (completely identical RNA-ligand complexes, but different PDB entries; only the one with best resolution was kept) and ligands with a quantitative estimate of drug-likeness (QED) score <0.4 were removed. This final data set comprises 92 RNAs in complex with drug-like ligands (Table S1). Physicochemical descriptors for the ligands were generated using MOE (Molecular Operating Environment (MOE); 2020.09; Chemical Computing Group ULC: 1010 Sherbrooke St. West, Suite #910, Montreal, QC, Canada, H3A 2R7, 2020, https://www.chemcomp.com/index.htm) (Table S1). All structures were visually inspected and structural corrections were implemented with MOE, if required. Ions present in X-ray structures were kept for all redockings except for the NMR versus X-ray analysis. Here, ions were removed from X-ray structures for comparison with the experimentally ion-free NMR structures (Table S2). The protonation states of the ligands were manually corrected using MOE for this analysis. A subset of 32 RNA-ligand complexes contained ions within the binding site (6 Å around the ligand), which was used to evaluate the influence of ions on the docking performance (Table S3). Ion-free structures were generated by removing all ions from the respective structures using PyMOL (PyMOL 2.4.1, Open-Source Build, Schrödinger, LLC, http://www.pymol.org).

### Cross-Docking Data Set

For cross-docking studies, a data set containing 43 X-ray structures of several natural as well as synthetic ligands across five RNA targets was assembled (Tables S4–S8). The PDB structures within a target group were aligned using PyMOL and further processed using MOE. All ions were kept as part of the target structures.

### Solvent Models

Different solvent models were used for analysis, namely solvent-free structures (*dry*) in which water molecules were removed, structures containing crystallographic water molecules (*wet*), and with solvation sites predicted by 3D-RISM^87^ (*rism*), a FlexX docking-based method reported previously (*waterdock_fxx*),^74^ and the machine learning-model GalaxyWater-CNN (*gw*).^63^

#### Wet

The generated RNA-ligand complexes were obtained from the Protein Data Bank (PDB)^38^ and solvent was not further modified.

#### Dry

The crystallographic water molecules were deleted from *wet* structures using PyMOL.

#### Rism

3D-RISM^87^ (three-dimensional reference interaction site model) belongs to the statistical mechanics methods and offers the opportunity to model complete water networks within the binding site in an efficient way. We employed the 3D-RISM method from MOE to build the solvated *rism* complex for docking. Here, the *dry* structure was first prepared via Quickprep without energy minimization to protonate and correct the structure for missing atoms^88^ while keeping atom coordinates comparable to other setups. The prepared structure was then subjected to solvent analysis of the binding site (7 Å around the ligand) maintaining a salt concentration of 100 mM to mimic the ion-rich environment surrounding RNA.^89^

#### Waterdock_fxx

In *waterdock_fxx*,^74^ a water molecule is repeatedly docked into the binding site using FlexX^82^ (FlexX Version 5.1.0, BioSolveIT GmbH, St. Augustin, Germany, https://www.biosolveit.de/FlexX) to identify possible hydration sites. The hydrated structures were generated using the *dry* structures as input and the cascade approach with a minimal distance to the ligand of 3.0 Å, in which the best-scoring water molecule is selected, all neighboring water molecules within 2.8 Å are deleted and the next best remaining water molecule is selected to proceed the method.

#### Gw

GalaxyWater-CNN^63^ is a 3D-convolutional neural network (CNN) able to predict solvation sites for apo or holo structures. It generates a water score map and implements different score cutoffs (34, 38, 42) to create hydrated structures (referred to as *gw34*, *gw38*, *gw42*). *Dry* holo structures were used for the predictions and all score cutoffs were applied for FlexX and HYDE redockings. For further analysis, including cross-docking studies, only *gw34* was generated and used for dockings, which retains the most water molecules.

### Docking Setups

Receptors containing no solvent, crystallographic or predicted water molecules (see above) were used for docking to investigate the impact of solvent inclusion on the docking performance. FlexX without or in combination with HYDE^83,84^ rescoring (hydescorer version 1.4.0, BioSolveIT GmbH, St. Augustin, Germany, https://www.biosolveit.de/HYDE), LeadIT^82^ (LeadIT version 2.3.2, BioSolveIT GmbH, St. Augustin, Germany) and GOLD^85,86^ (Hermes version 2025.1.0 for redocking studies and 2025.3.0 for X-ray vs NMR analysis, The Cambridge Crystallographic Data Centre, Cambridge, United Kingdom, https://www.ccdc.cam.ac.uk/solutions/software/gold/) were applied for dockings. The docking setups except for HYDE were previously validated for RNA-ligand dockings.^17,25,90,91^

#### FlexX

Ligands from the RNA-ligand complex structures in sd-file format were used as input for docking. Protoss^92^ is implemented in FlexX for automated protonation and tautomerization. Twenty poses per ligand were generated with only the top pose being subjected to root-mean-square deviation (RMSD) calculation using obrms from Open Babel^93^ (Open Babel version 3.1.1, 2021, http://openbabel.org).

#### HYDE

The 20 output poses of FlexX docking were rescored using the HYDE scoring function The subsequent top-scoring pose was used for RMSD calculation using obrms.

#### LeadIT

Structure preparation and docking was conducted through the graphical user interface using default settings. The cocrystallized ligand was used for binding site definition (6.5 Å around ligand). Protonation was performed by Protoss, which is included in LeadIT. Water molecules with at least three interactions with the binding site and/or ligand were considered for docking having fully specified water coordinates. Default settings for docking used the enthalpy–entropy hybrid approach with 200 iterations per placement and fragmentation. Target-ligand clashes had a maximum allowed overlap volume of 2.9 Å^3^ and intraligand clashes a clash factor of 0.6 including hydrogen atoms in the internal clash tests. RMSD calculation was performed within LeadIT for the top pose.

#### GOLD

The input files for docking were configured through the graphical user interface of Hermes. Parameters for an exemplary GOLD docking configuration file are provided in the Supporting Information (Table S9). In GOLD docking, up to 24 water molecules are tolerated. The number of water molecules in solvated complex structures was limited by removing water molecules over 6 Å away from the ligand using PyMOL In case, too many water molecules remained, the cutoff distance around the ligand was reduced in 0.5 Å steps, and under 4.0 Å in 0.1 Å steps until the acceptable number of water molecules was reached (Table S1). Protonation of the structures was performed within the GOLD Wizard, followed by extraction of the water molecules and the ligand, which are later reimplemented. The extracted ligand was used for binding site definition, which implies all atoms within 6 Å around the ligand. Ten GA runs were conducted and the “allow early termination” was disabled to search a larger set of ligand poses. GoldScore was used as the scoring function, which was previously validated for RNA targets.^25,91^ The default settings were used for water molecules, enabling the toggle and spin option to allow inclusion or displacement of water molecules as well as to optimize water orientations. The top pose was selected for RMSD calculation within GOLD.

#### Figures

Figures were created with PyMOL, violine plots with RStudio (RStudio 2023.12.1 + 402 “Ocean Storm”, Posit team (2024). RStudio: Integrated Development Environment for R, Posit Software, PBC, Boston, MA. URL http://www.posit.co/).

## Results and Discussion

### Redocking

Using a data set of 92 RNA—drug-like ligand complexes, different combinations of solvent models and docking tools were tested. FlexX, GOLD and LeadIT were previously reported to be suitable for RNA-ligand docking. FlexX with HYDE rescoring (referred to as HYDE) was additionally employed as an alternative to FlexX docking. These setups were combined with five different solvent models—without solvent (*dry*), with crystallographic water (*wet*), and with predicted solvation sites by 3D-RISM (*rism*), waterdock_fxx (*waterdock_fxx*) and GalaxyWater-CNN (*gw*). Ions were kept as part of the target site for the generation and docking of all solvated RNA structures. GalaxyWater-CNN can operate with three different score-cutoffs for the predicted water molecules, generating three different hydrated structures (*gw34*, *gw38*, *gw42*). Redockings using FlexX and HYDE with all three *gw* structures showed the highest docking accuracy for *gw34* (Figure S1 and Table S1) which was proceeded in this study. GalaxyWater-CNN with the score cutoff of 34 (*gw34*) includes the highest number of water molecules. A redocking was classified as successful when docking poses exhibited a RMSD value ≤ 2.0 Å with respect to the native binding mode. For FlexX, redocking success rates were only slightly affected by the inclusion of different solvent models with success rates spanning 71–77% (Figure 1A, and Table 1) being in line with a previous study for proteins^74^ and confirming reasonable pose prediction accuracy for RNA-targets.^17^ FlexX involves water molecules with at least three potential interactions with the binding site in pose generation, while remaining water molecules are displaceable during docking. In case of HYDE rescoring, water molecules participating in a minimum of three hydrogen bond interactions with either the binding site or the ligand are retained for pose optimization and rescoring. Similarly to FlexX, HYDE did not benefit from the inclusion of solvation sites, but showed an overall weaker performance of around 50% correctly predicted binding modes (Figure 1B and Table 1), suggesting that HYDE rescoring is not to be preferred over FlexX scoring for RNA-ligand docking. LeadIT redocking performances demonstrated to be more sensitive toward the used solvent model. The combination of LeadIT and *rism* achieved the overall highest observed success rate of 87%, followed by 82% with *gw34*, while the *dry* setup only reached 73% (Figure 1C and Table 1). By default, LeadIT considers water molecules with at least three interactions with the binding site and/or ligand and fully specified water coordinates. Consequently, LeadIT redockings uses constrained water molecule orientations, indicating why LeadIT is more sensitive toward solvent inclusion. Likewise, GOLD was more sensitive to the implemented solvent compared to FlexX and HYDE. The difference in docking performances between solvated (*rism* 76%; *wet/gw34* 73%) and *dry* (67%) setups was also observable in GOLD redockings (Figure 1D and Table 1) contrary to a previously reported study.^25^ By default, GOLD considers each water molecule present in the binding site. During docking, GOLD retains or displaces water molecules (=toggle option) depending on the estimated free-energy change when a water molecule is transferred from the bulk solvent to its binding site. In addition, the orientation of the water molecules is automatically optimized (=spin option).^94^

**Table 1:** Redocking Success Rates in % Defined as Predicted Binding Modes with RMSD ≤ 2.0 Å Compared to Experimental Structure for All Resolutions (Based on 92 Entries), High-Resolution (≤2.0 Å, 29 Entries), Medium-Resolution (2.0–3.0 Å, 44 Entries), Low-Resolution (> 3.0 Å, 8 Entries, and 11 NMR Structures)^a^

docking tool	resolution	*dry*	*wet*	*rism*	*waterdock_fxx*	*gw34*
FlexX	all	72	75	77	71	73
	≤2.0 Å	86	86	90	86	90
	2.0–3.0 Å	77	84	84	75	77
	>3.0 Å and NMR	37	37	42	42	37
HYDE	all	50	50	52	52	52
	≤2.0 Å	48	41	52	45	69
	2.0–3.0 Å	61	66	59	61	50
	>3.0 Å and NMR	26	26	37	42	32
LeadIT	all	73	75	87	76	82
	≤2.0 Å	90	97	97	76	100
	2.0–3.0 Å	82	82	93	86	89
	>3.0 Å and NMR	26	26	58	53	37
GOLD	all	67	73	76	72	73
	≤2.0 Å	76	79	79	72	79
	2.0–3.0 Å	73	82	86	80	82
	>3.0 Å and NMR	42	42	47	53	42

a Visualization as violine plots is shown in Figure 1 and in Figure S3. Raw data found in Table S1.

**Figure 1:**
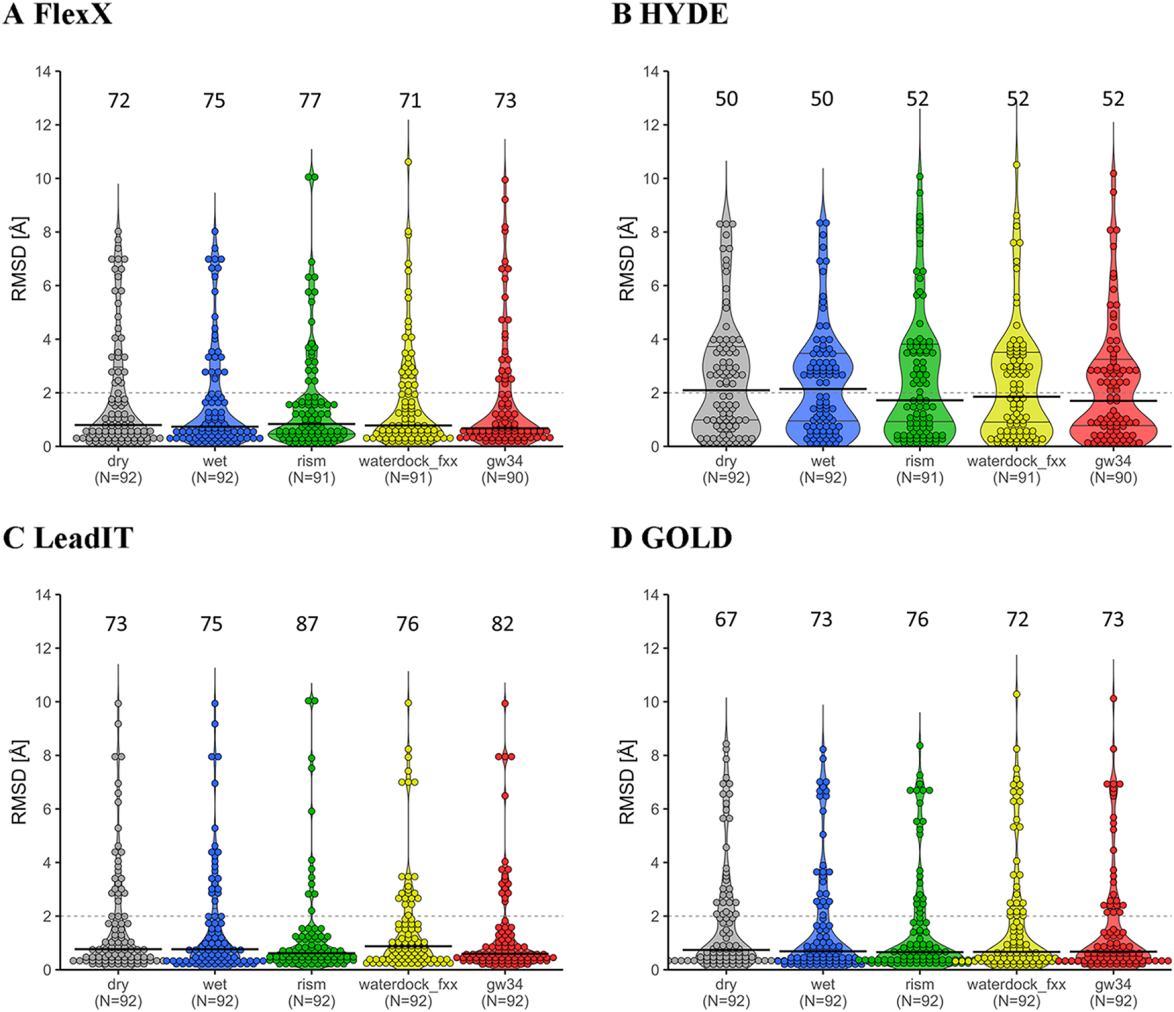
Redocking RMSD values across different solvent models summarized as violin plots for *dry* (gray), *wet* (blue), *rism* (green), *waterdock_fxx* (yellow) and *gw34* (red) dockings. Results are grouped by docking tool: (A) FlexX, (B) HYDE, (C) LeadIT and (D) GOLD. Distribution centers and variability are indicated by median (bold line) as well as first and third quartiles (thin lines). An RMSD value of 2.0 Å (dotted line) is used as cutoff for successful redockings and success rates in % are given above the violines. Raw data shown in Table S1.

### Structural Analysis

Redocking RMSD values across the different docking tools and solvent models showed no strong correlations, but indicated some reoccurring trends (Table S1 and Figure S2). From a ligand perspective, a lower number of rotatable bonds, low or no charge, and a lower molecular weight resulted in higher pose prediction accuracies, while no trend was found for other common physicochemical descriptors (Table S1). Thus, successful redockings across docking tools and solvent models included mostly rigid, neutral and small ligands (exemplarily shown for hypoxanthine in complex with the guanine riboswitch, Figure 2A). Most well performing targets consisted of riboswitches bound to their natural ligands or derivatives thereof. Cases in which all docking tools failed were mostly represented by NMR structures or targets including big and flexible ligands. Nonriboswitch targets which do not natively bind a small molecular weight ligand, like the hepatitis C virus (HCV) internal ribosome entry site (IRES), human immunodeficiency virus (HIV)-1 transactivation response (TAR) element, human RNAs or artificial aptamers, accumulated in the group of unsuccessful redockings. These RNAs often share similar binding site properties in form of bulges and bear solvent-exposed regions (exemplarily shown for the SMN2 exon7 in complex with a risdiplam-like synthetic ligand, Figure 2B). Regions of unpaired nucleobases, like in riboswitch aptamers, are often considered to form more complex binding sites with a higher information content^95,96^ associated with improved ligandability. A higher structural complexity of the ligand binding site might also correlate with a better docking performance. However, the used data set is biased toward riboswitches and further studies (and RNA-ligand complex structures in general) are needed to elucidate this hypothesis. Lastly, there were no hints indicating challenging structural features specific for a docking tool. Only a few cases in which one docking tool (mostly LeadIT or GOLD, Table S1) outperformed the others were noticed (exemplarily shown for the thiamine pyrophosphate (TPP)-RS—fragment complex, Figure 2C). This agreed with the previous observation that successful redockings are usually found across different tools (Figure S2). Similarly, there was a low number of occurrences in which the same solvent model improved the docking performance across the tools while others did not. Different solvent models enhanced redockings for different tools in a target-specific manner (exemplarily shown for the tetrahydrofolate RS, Figure 2D).

**Figure 2:**
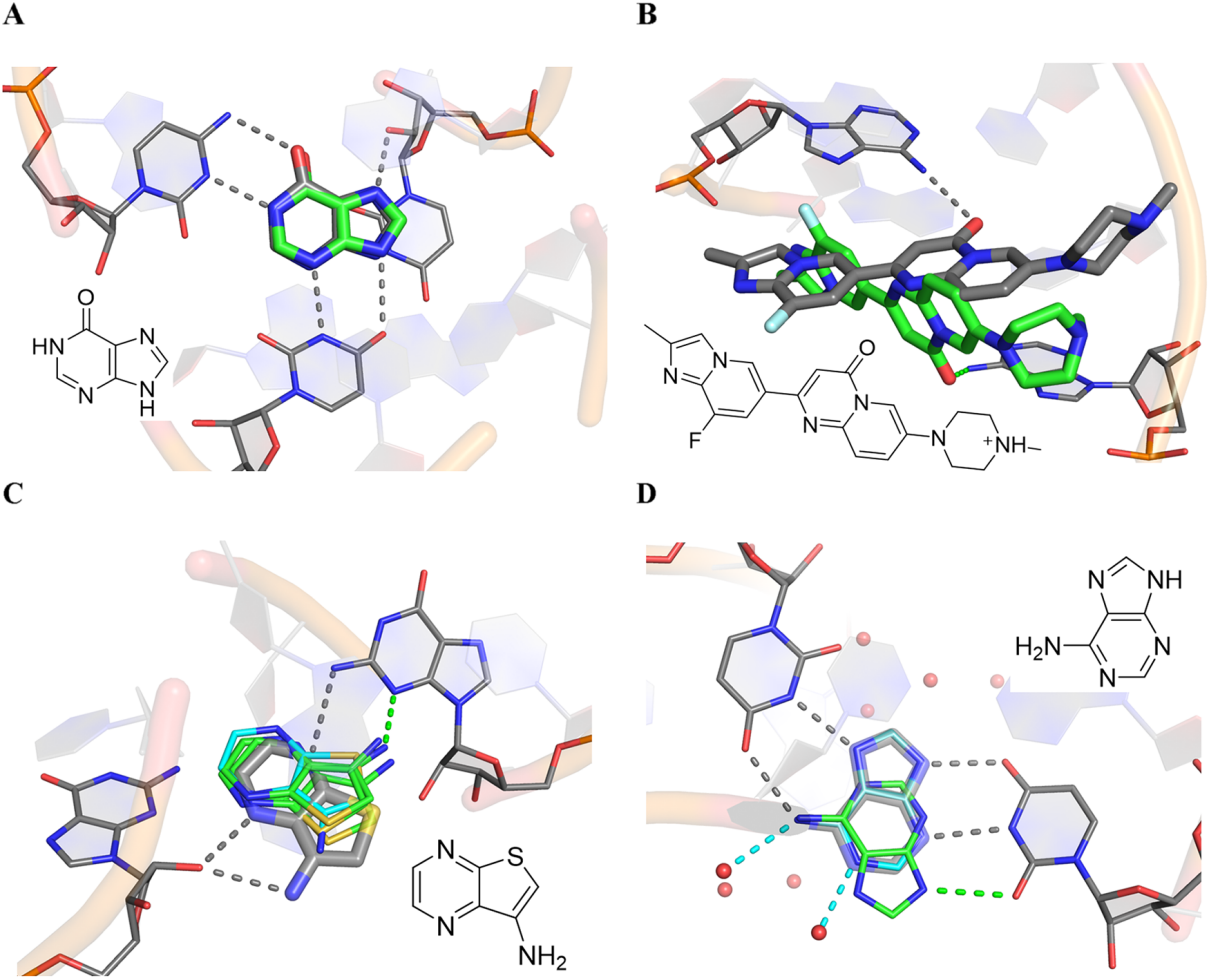
Examples for redocking with structures performing consistently well (A) or bad (B) over different docking tools and solvent models. Exemplary structure in which one docking tool (GOLD) outperforms the others (C) or solvent-information improves redocking compared to *dry* (D). X-ray structures are shown in gray and representative docking poses in green unless otherwise stated. (A) X-ray structure and representative docking pose (FlexX, *dry*) of a guanine riboswitch in complex with hypoxanthine (PDB 2EET,^97^ RMSD = 0.2 Å), (B) NMR structure and representative docking pose (FlexX, *dry*) of the RNA duplex formed by the 5′-end of U1snRNA and the 5′-splice site of SMN2 exon7 (PDB 6HMO,^98^ RMSD = 4.0 Å), (C) X-ray structure and multiple docking poses of the TPP riboswitch in complex with a fragment (PDB 4NYC,^99^ *dry*, FlexX RMSD = 2.8 Å, HYDE RMSD = 2.7 Å, LeadIT RMSD = 2.8 Å, GOLD RMSD = 1.5 Å, all docking poses are shown in green except for GOLD which is in cyan) and (D) X-ray structure and representative docking poses of the tetrahydrofolate riboswitch in complex with adenine (PDB 4LW0,^100^ HYDE *dry* RMSD = 3.2 Å in green, *wet* RMSD = 0.1 Å in cyan). Dotted lines represent polar interactions.

### Resolution Dependency

The compiled data set of 92 RNA-ligand complexes includes 11 NMR structures and 81 crystal structures comprising 29 high-resolution (≤2.0 Å), 44 medium-resolution (2.0 Å–3.0 Å), and 8 low-resolution (>3.0 Å) structures (Table S1). We hypothesized that docking performance across different solvent models may also depend on the resolution of the RNA-ligand complex structures. NMR structures were also assigned to the low-resolution category, as both NMR structures and low-resolution X-ray structures contain no solvent site information. A pronounced decrease in success rates was observed in high- over medium- to low-resolution structures for most docking tools. (Table 1 and Figure S3). As an exception, HYDE (and partially GOLD) redocking success rates improved for medium-resolution structures compared to high-resolution (Figure S3 and Table 1). In general, high- and medium-resolution structures benefited slightly from the inclusion of explicit water molecules in the binding site from *wet*, *rism* and *gw34* compared to *dry* dockings (Table 1). At low resolutions or in NMR structures, solvent site information is missing in *wet* structures, thus, *wet* and *dry* structures were identical. Solvent models were partially able to counteract the missing information and the decrease in docking performance. Notably, *rism* and *waterdock_fxx* retained some of the accuracy in contrast to the other solvent models across all docking tools (Figure S3 and Table 1). *Waterdock_fxx* might be useful for “salvaging” low-resolution or NMR structures, while *rism* can be regarded as a robust solvent model supporting acceptable docking performances across all resolutions relative to the other models (Table 1). Overall, redocking performances benefited from inclusion of solvent models and the extent of the observed effect seemed to be resolution-dependent.

More than half of the low-resolution group consisted of NMR structures, which showed deteriorated success rates when comparing to low-resolution (>3.0 Å) crystal structure redockings (Table S1). Consequently, the previous observed deterioration might mainly be caused by implementing NMR structures. For a direct comparison, three RNA-ligand complexes, which have an X-ray as well as NMR structure available in the PDB were selected. These were used to evaluate the impact of experimental origin on the redocking RMSD value for *dry* setups independent of solvent information. NMR and X-ray structure pairs were found for the prequeuosine_1_ (PreQ_1_) class I riboswitch (RS) in complex with PreQ_1_, the HCV IRES subdomain IIa in complex with two similar synthetic ligands, and the theophylline aptamer with theophylline as the ligand (Figure 3A–C). Moreover, we evaluated the usage of cryo-EM as an emerging technique by comparing the X-ray and cryo-EM structures of the TPP RS in complex with TPP (Figure 3D). For comparability of X-ray and NMR structures, ions were deleted from X-ray structures. The superimpositions of X-ray and NMR structures showed similar interaction patterns and a stable binding site across the different NMR conformers (Figure 3A,C) except for the HCV IRES (Figure 3B). The synthetic ligands of the HCV IRES differ in the size of the oxygen-containing ring (tetrahydrofuran in the NMR complex, tetrahydropyran in the X-ray structure). The higher flexibility of the HCV IRES compared to the other RS and the fact, that these ligands induce a conformational change upon binding,^101,102^ might justify the observed structural differences (Figure 3B). The cryo-EM structure of the TPP RS aligns very well with the corresponding X-ray structure including the position of the ligand, binding site residues and ions as well as the interaction pattern (Figure 3D). In most cases, redocking success rates were equal or better for X-ray structures compared to NMR structures (Tables 2, and S2). Depending on the frame used for docking with NMR structures, redocking RMSD values varied. While good poses often correlated with lower RMSD-values for the respective NMR frames, the best scoring solution not necessarily had the lowest RMSD value (Table S2). For the HCV IRES and the theophylline aptamer, successful redockings were only obtained when using LeadIT and GOLD setups with X-ray structures. The failure of FlexX (and HYDE) redockings of the HCV IRES X-ray structure was attributed to wrong protonation of the ligand, as described previously.^16,103^ The automated protonation by Protoss within FlexX during docking cannot be customized in contrast to GOLD and LeadIT dockings. Here, the right protomer (positively charged 2-amino benzimidazole, total charge +3) was implemented for redockings, which explains the improved results. The success of cryo-EM redockings depended on the docking tool used, but was comparable to X-ray redocking performances. The different behaviors in redockings might be caused by a combination of size, flexibility and complexity of the RNA-binding sites and the ligands. Despite the low number of methodological complex-structure pairs for direct comparison, these case studies suggest that the probability of successful redocking decreases when using NMR structures compared to X-ray, agreeing with previous studies.^17,25,79^ However, further test cases are required for statistical significance. The wide range of RMSD values when including all NMR frames also indicate that it might be reasonable to use an ensemble of NMR conformers,^25^ if no X-ray structure is available. However, frame selection from an ensemble is not trivial, as best RMSD values did not directly correlate with highest docking scores. In addition, the emerging technique of cryo-EM might be a good alternative to X-ray crystallography for structure elucidation and subsequent docking approaches (Figure 3D, Tables 2 and S2). Further, ions play an important role in RNA structures^104–109^ and knowledge about their positions may guide SBDD, which is not applicable for NMR structures.

**Figure 3:**
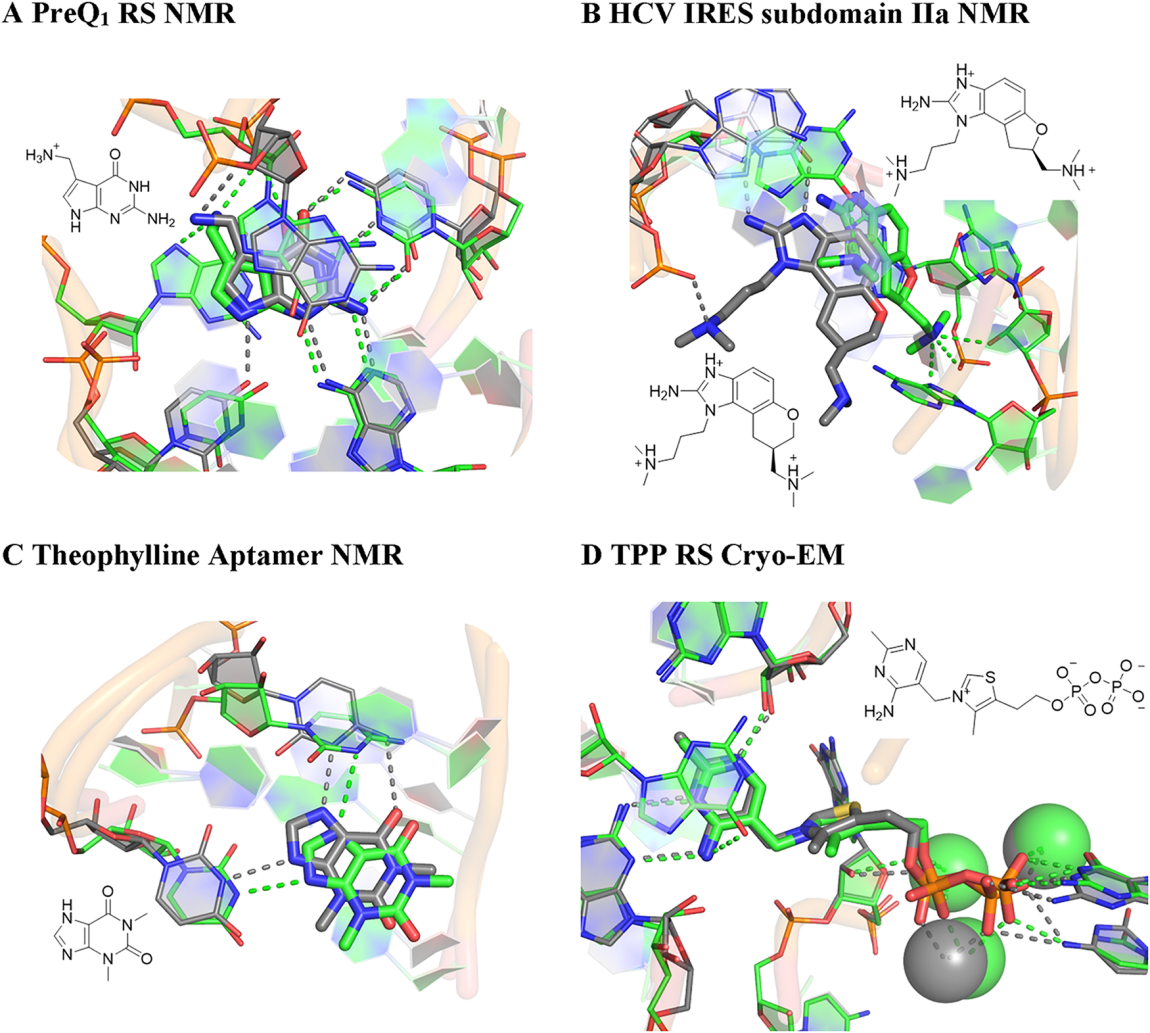
Superimposition of ion- and solvent-free X-ray structures and NMR structures (first conformer shown) for (A) PreQ_1_ class I RS (PDB 3Q50,^110^ 2L1 V,^111^ RMSD RNA binding site (residues within 8 Å around the ligand) = 0.9–1.1 Å, RMSD ligand = 0.7–1.0 Å), (B) HCV IRES subdomain IIa (PDB 3TZR,^101^ 2KTZ,^112^ RMSD RNA = 2.2–3.7 Å, RMSD ligand = 6.9–7.6 Å) and (C) theophylline aptamer (PDB 8D28,^113^ 1EHT,^114^ RMSD RNA = 1.9–3.6 Å, RMSD ligand = 0.6–1.8 Å) as well as superimposition of solvent-free X-ray structure and cryo-EM structure of (D) TPP RS (PDB 2GDI,^115^ 9C6K,^116^ RMSD RNA = 0.6 Å, RMSD ligand = 0.9 Å) (Table S2). Crystal structures are shown in gray, and the NMR structures and the cryo-EM structure in green. Dotted lines represent polar contacts and spheres represent magnesium ions.

**Table 2:** Comparison of Redocking RMSD Values in Å for *Dry* Docking with FlexX, HYDE, LeadIT and GOLD Using X-ray, Solution NMR (All Available Conformations Were Used, RMSD Range Given) or cryo-EM Structures of the PreQ_1_ RS, HCV IRES Subdomain IIa, Theophylline Aptamer or TPP RS

docking tool	method	PreQ_1_ RS^a^	HCV IRES^b^	theophylline aptamer	TPP RS
FlexX	X-ray	0.7	5.3	3.0	2.0
	NMR	0.4–0.9	6.2–9.6	3.1–3.9	
	Cryo-EM				2.0
HYDE	X-ray	0.6	5.2	4.1	5.1
	NMR	0.5–2.7	4.7–9.4	3.0–3.7	
	Cryo-EM				2.2
LeadIT	X-ray	0.5	2.1	0.7	1.7
	NMR	0.3–1.0	6.5–9.7	3.4–4.2	
	Cryo-EM				5.4
GOLD	X-ray	0.5	1.0	0.3	2.1
	NMR	0.4–0.7	2.8–7.7	1.6–3.8	
	Cryo-EM				2.5

a Binding site residues of X-ray and NMR structure differed in two nucleotides.

b X-ray and NMR cocrystallized ligands were slightly different in their structures. Raw data shown in Table S2.

### Influence of Ions

The redocking data set contained 32 RNA-ligand complexes, which have at least one ion within the binding site (6 Å around the ligand) (Table S1). Ions play an important role in RNA structures. In general, positively charged ions diffuse around the RNA due to the negatively charged backbone forming a diffuse ion atmosphere. But ions can also bind to specific sites stabilizing certain RNA conformations, promoting conformational changes, facilitating binder recognition or mediating RNA stability, among others. Determination of ion binding sites is not trivial, neither experimentally nor computationally.^104,106–109,117–119^ Usually high-resolution X-ray structures are used to determine ion sites. However, these crystallographic methods impose high salt concentrations differing from physiological conditions. Thus, crystallographically determined ion sites might be misleading when applied on rational design approaches. In addition, prediction or refinement of ion sites by molecular dynamics simulation can be demanding not only due to long convergence times of ions.^118,120^ The importance of ions for RNA structures suggests that binding site ions might affect docking outcomes. The subset of 32 RNA-ligand complexes was used to investigate the influence of ions on docking performances including the five water models and FlexX as the docking tool. FlexX was selected due to its highly automated workflow and its consistent performance in previous analyses (Table 1). Most of the redockings in which binding site ions were removed showed a deteriorated success rates in comparison to redockings containing the ions (Table 3, Figure S4 and Table S3). While *dry* structures experienced the biggest decrease in docking performance, followed by *waterdock_fxx*, *rism* maintained the highest docking accuracy of 78% (Table 3). Solvent models *wet*, *rism* and *gw34* were able to counteract the lack of ions and the decline in success rate, similar to what was observed for low-resolution structures (Table 1 and Figure S3). The observed higher success rate for ion-containing redockings can arise from beneficial ligand-ion interactions guiding toward the native binding mode or spatial restriction ions possess on pose prediction. In a real-world drug discovery project, all information regarding conserved ion binding sites may guide SBDD. Even more than water molecules, ions can be considered as part of the binding site eventually interacting with the ligand. However, in specific cases ligands can also replace less tightly bound ions.^121^

**Table 3:** Comparison of FlexX Redocking Success Rates in % Defined as Predicted Binding Modes with RMSD ≤ 2.0 Å Relative to Experimental Structure for a Subset of 32 RNA-Ligand Complexes with Ions and without Ions^a^

setup	*dry*	*wet*	*rism*	*waterdock_fxx*	*gw34*
with ions	72	78	78	72	69
ion-free	53	69	78	59	63

a Visualization as violine plots and redocking RMSD values are shown in Figure S4 and Table S3, respectively.

### Protonation States

Ligand protomers and tautomers can influence docking performance. Different docking tools imply distinct protonation methods. FlexX and HYDE protonate via Protoss^92^ dynamically during the docking and scoring process. For LeadIT and GOLD, protonation is defined from the ligand’s input structure. In 37 out of 92 entries some protomers differed between docking outputs, partially in a solvent-dependent manner (Table S1). For HYDE protonation of pyrimidine and imidazole moieties and deprotonation of some lactam moieties (in proximity to binding site ions) were occasionally found. In some of these cases the inaccurate docking might therefore be attributed rather to protonation via Protoss^92^ than the HYDE scoring function itself. However, the automated protonation during docking with FlexX and HYDE rescoring does not allow for user intervention to correct misannotated protonation states. GOLD in some cases did not protonate aliphatic amines, while LeadIT’s protonation was the most accurate. Still, GOLD achieved similar high docking success rates like FlexX and LeadIT suggesting that these protonation failures did not hamper success rates (Table 1). All docking tools used standard protomers and tautomers of the nucleobases. While there is no rare protomeric or tautomeric state of nucleobases reported to be specifically targeted in literature, local environments can stabilize rare states which holds the potential for selective targeting.^103,122–124^

### Cross-Docking

Inclusion of different solvent models can improve redocking accuracy, especially for low-resolution and NMR structures (Table 1). Successful redocking is a prerequirement to validate the setup used for subsequent structure-based VS. However, including a higher number of water molecules narrows the docking sampling space biasing the docking toward the native ligand and binding mode. Although, this is associated with an improved pose prediction accuracy in redocking, limiting ligand space and orientation may contribute to failures in cross-docking and subsequent prospective VS.^67,70,71,74^ Despite this, the additionally introduced hydrogen bonds by water molecules may shield electrostatic interactions helping to generate near-native docking poses.^25^ Therefore, cross-docking studies for five RNA targets and a total of 43 ligands were conducted. Ions were kept for solvent generation and dockings. The previously used solvent models *dry*, *wet*, *rism*, *waterdock_fxx* and *gw34* were combined with FlexX docking, which showed most consistent performance in a highly automated workflow (Table 1). Especially this accounts for medium-resolution structures, which dominate this cross-docking data set (Tables S4–S8). In total, 43 crystal structures were used for cross-docking analysis comprising the theophylline aptamer, guanine, FMN, PreQ_1_ and TPP RSs (Tables 4, and S4–S8). For the theophylline aptamer and guanine RS, ligands were mostly derived from the natural binder, thus implying a small and planar moiety with changing substituents. The ligands of the FMN RS contained either the flavin core while substituents differed, or were inferred from ribocil. PreQ_1_ RS ligands can be split into three PreQ_1_-like ligands and five ligands being dibenzofuran derivatives. Half of the TPP RS ligands derived from TPP, while the other half belonged to structurally distinct fragments, and a fragment-derived lead. Both re- and cross-dockings of the theophylline aptamer benefited from solvent information. Especially, *rism* as the solvent model significantly improved the success rate reaching 40% versus 10 and 20% for *dry* re- and cross-dockings, respectively (Tables 4, and S4). In contrast, all redockings of the guanine RS were successful, while cross-dockings performed best with *dry* having 95% success rate, followed by *wet* with 86%. Inclusion of solvent for cross-dockings might have introduced too much ligand bias preventing correct pose prediction for distinct ligands (Tables 4, and S5). FMN RS redockings improved the most with *wet*, *waterdock_fxx* and *gw34* setups (50%) relative to *dry* and *rism* (38%). The corresponding cross-docking success rates were similar across all solvent models maintaining around 30% success rate (Tables 4, and S6). Thus, the general decrease in cross-docking performance was not attributed to solvent inclusion in this example. In case of the PreQ_1_ RS, the best solvent model for redockings (*rism*, 75%) differed from the one for cross-dockings (*wet*, *gw34*, 38% *versus rism*, 32%) (Tables 4 and S7). Contrary, the most successful redocking solvent models (*rism*, 57%, *gw34*, 64%) were able to indicate the best cross-docking setups (*rism*, 27%, *gw34*, 24% *versus dry*, 20%) for the TPP RS (Tables 4 and S8). The selected RNA-ligand structures within the same target class share identical binding site residues. However, some complexes of the PreQ_1_ and TPP RS show ligand-induced conformational changes of specific residues emphasizing the intrinsic flexibility of RNA. For PreQ_1_, residue C15 varies in its conformation and for the TPP RS residue G72, or these residues were abasic by experiment (Figure S5). Cross-dockings can be affected by the different residue orientations. Therefore, PreQ_1_ and TPP RS dockings were grouped based on similar binding site conformations (Table 4, Figure S5, Tables S7 and S8). By doing so, the effect of explicit solvent on re- and cross-docking accuracy can be interpreted independently from these conformational changes. The docking performances within these groups do not necessarily cover the best RMSD values relative to dockings outside the groups, but for several ligands only good poses were found for the same binding site conformation (Tables S7 and S8). For the PreQ_1_ RS, *wet, rism* (43%) and *gw34* (50%) were found to be the best solvent model when summarizing all cross-dockings within the defined groups. These solvent models partially overlapped with best redocking setups (Tables 4 and S7). Similarly, for TPP RS, *rism* and *gw34* performed best in redockings, while *rism* (38%) was found to also produce the best cross-docking poses. Notably, *dry* cross-dockings seemed to generally produce inferior re- and cross-docking results for the TPP RS groups (Tables 4 and S8). Some fragment-sized TPP RS ligands might also be less affected by the different conformations as these do not interact with G72. Target-specific effects of individual solvent models became less pronounced when reand cross-docking results were summarized across all five targets and only including the defined groups for the PreQ_1_ and TPP RS. *Rism* performed best with a 50% success rate, followed by *wet* and *gw34* (47%) for cross-dockings. This is in accordance with redockings where *rism* (60%) and *gw34* (58%) showed the highest success rates in comparison to *dry* (49%) when summarizing across all targets (Table 4). However, this beneficial effect of the inclusion of explicit water molecules was less pronounced for cross-dockings compared to redockings.

**Table 4:** Redocking and Cross-Docking (Number of Docking Data Points) Success Rates in % Defined as Predicted Binding Modes with RMSD ≤ 2.0 Å Compared to Crystal Structure Using Different Solvent Models and FlexX as the Docking Tool for Five Targets and Groups within a Target Class Containing Multiple Conformers

	target					
	theophylline aptamer		guanine RS		FMN RS	
solvent model	redocking (5)	cross-docking (20)	redocking (8)	cross-docking (56)	redocking (8)	cross-docking (56)
*dry*	20	10	100	95	38	29
*wet*	40	25	100	86	50	30
*rism*	40	40	100	82	38	30
*waterdock_fxx*	40	20	100	80	50	29
*gw34*	20	25	100	82	50	30

	PreQ_1_ RS			TPP RS			all targets	
solvent model	redocking (8)	cross-docking all (56)	cross-docking groups^a^ (14)	redocking (14)	cross-docking all (182)	cross-docking groups^a^ (32)	redocking (43)	cross-docking groups^a^ (178)
*dry*	63	34	36	36	20	16	49	46
*wet*	63	38	43	43	24	25	56	47
*rism*	75	32	43	57	27	38	60	50
*waterdock_fxx*	50	34	36	50	20	28	56	44
*gw34*	50	38	50	64	24	28	58	47

a A group refers to a similar conformation of the binding site residues. For PreQ_1_ residue C15 and for TPP RS mainly G72 undergoes conformational changes induced by different ligands or the nucleobase is missing (Figure S5). Detailed redocking and cross-docking results for all structures are shown in Tables S4–S8.

This cross-docking analysis underlined the potential benefit of including solvent information on the docking performance. Generally, cross-docking success rates were deteriorated when compared to the corresponding redockings, but the decrease was in most cases independent of solvent inclusion and likewise impaired *dry* dockings. The improvements of hydrated redockings translated partially to cross-dockings. But the choice of the solvent model was target-dependent. The solvent model *rism* was found to be a robust solvent model across re- and cross-dockings and across all resolutions (Tables 1 and 4). The different binding site conformations of two targets induced by different ligands emphasized RNA dynamics as additional known challenge in RNA-ligand docking. Even small conformational changes of a single residue can affect docking performances dramatically. Summing up, redocking studies including different solvent models may help to identify docking tool—solvent model combinations well suited for cross-dockings and subsequent VS for a given target.

### Further Considerations

The four docking tools used, FlexX, HYDE, LeadIT and GOLD, imply different solvent handling methods. While FlexX, HYDE and LeadIT select water molecules based on the number of interactions with the binding site or the ligand, GOLD estimates the free-energy change of a water molecule when transferring from the bulk solvent to the binding site. In general, the study was conducted with automated workflows to observe solvent effects on docking performance, thus minimizing human bias. In contrast, in real-world drug discovery applications, all available information regarding key interactions and water molecules may be implemented. The inclusion of structural water molecules was found to be beneficial in RNA-ligand dockings reported previously.^13,17,25,79,80^ The beneficial effect of including explicit solvent could be further enhanced by incorporating improved solvation strategies, scoring functions or novel modalities into docking workflows. A solvent prediction model showed promising results in identifying solvation sites in nucleic-acid-ligand complexes by employing statistical information of water molecules around nucleotides and a hydrogen-bonding-optimized scoring function.^64^ Computationally more expensive techniques, like SPAM^125^ or WaterMap,^126,127^ derive hydration sites from explicit solvent molecular dynamics simulations and provide additional thermodynamic information.^53,125^ Implementing these refined solvent sites might be beneficial for RNA targets, although SPAM did not perform better than common solvent models in some test cases for proteins.^74^ In this study, additional challenges like the intrinsically dynamic nature of RNA^21,22,118,120^ or the impact of structurally relevant or indirectly interacting ions^104–109^ on docking were neglected, to only focus on solvent effects. However, the impact of target conformations became already evident in the cross-docking study (Table 4 and Figure S5). Moreover, the influence of ions on the docking performance became apparent in a subset of the redocking data set (Tables 3, S3 and Figure S4), but further studies are needed to identify clear correlations.

## Conclusion

In this work, the influence of explicit solvent inclusion on RNA-ligand docking performance was examined using four docking setups in combination with five solvent models. Overall, acceptable redocking performances for FlexX, GOLD and LeadIT were found in line with previous studies. Only HYDE rescoring seemed to be less suitable for several RNA targets compared to FlexX docking alone using the empirical FlexX scoring function. The implementation of explicit solvent was most beneficial for LeadIT and GOLD setups. Redockings were most successful when small and rigid ligands were docked into complex binding sites. The solvent model *rism* showed most robust results across all resolutions meaning that no high-resolution structure may be necessary. Similarly to *rism*, *waterdock_fxx* demonstrated high resilience at low-resolution and NMR structures. In general, NMR structures were found to decrease pose prediction accuracy compared to X-ray structures. The cryo-EM example of the TPP RS indicated the potential of this emerging technique as an alternative to X-ray crystallography, although further validation will be required for a meaningful comparison. Analysis of ion-containing and ion-free redockings indicated beneficial effects when including ions. Similar to structures with low resolutions, solvent models were able to partially counteract the deteriorated success rates arising from the lack of ions. Moreover, cross-docking studies were conducted with five RNA targets cocrystallized with different ligands. Implying solvent information during cross-dockings improved success rates compared to *dry* setups in most cases. In addition, hydrated redockings were found to be predictive for the corresponding hydrated cross-dockings in a target-dependent manner. From these cross-dockings, the other main challenge for RNA-ligand docking became evident—RNA dynamics. While not being the focus of this manuscript, conformational changes of binding site residues also had an impact on cross-docking accuracy independent of the used solvent models.

Summing up, the benefit of solvent inclusion is highly target-specific and resolution-dependent and has to be validated for the individual target of interest. If no experimental solvent information is available, *rism* demonstrated a robust improvement for reand cross-dockings. Beyond solvation, major challenges in RNA docking persist, including the accurate modeling of ligand-induced conformational changes or incorporation of ions. This work addresses only one isolated challenge in RNA SBDD, the implementation of explicit water molecules in RNA-ligand docking. In doing so, it contributes a small but meaningful piece to the larger puzzle that must be assembled to enable the full potential of rationally targeting RNA with small molecules.

## Supplementary Material





## Data Availability

The lists of PDB entries used for the RNA-small molecule re- and cross-docking data sets as well as the RMSD raw data are provided in the Supporting Information (XLSX). The tools FlexX, HYDE, LeadIT and GOLD were employed for molecular dockings using the versions mentioned in the Materials and Methods part. The solvent model *rism* was created *via* the 3D-RISM method of the cited MOE version. The scripts along with usage documentation of *waterdock_fxx* (10.1021/acs.jcim.5c02352) and Galaxywater-CNN (https://github.com/seoklab/GalaxyWater-CNN, Web server: https://galaxy.seoklab.org/cgi-bin/submit.cgi?type=GWCNN_INTRO) are freely available.

## Notes

Figures including additional violine plots for *gw*, resolution dependency and ion analyses as well as correlation scatter plots for the redockings and visualizations of conformational changes for the cross-dockings data set: (Figures S1–S5); additional references 128–132 (PDF)
Additional tables representing data set information as well as detailed re- and cross-docking results of the different analyses (Tables S1–S9) (XLSX)

## Abbreviations

3D-RISM: three-dimensional reference interaction site model
C: Cytidine
CNN: convolutional neural network
EM: electron microscopy
FDA: U.S. Food and Drug Administration
FMN: flavin mononucleotide
G: guanosine
HARIBOSS: Harnessing RIBOnucleic acid–Small molecule Structures
HCV: hepatitis C virus
HIV: human immunodeficiency virus
HTS: high-throughput screening
IRES: internal ribosome entry site
MOE: Molecular Operating Environment
ncRNA: noncoding RNA
NMR: nuclear magnetic resonance
PDB: protein databank
PreQ_1_: pre-queuosine1
QED: quantitative estimate of drug-likeness
RMSD: root-mean-square deviation
RNA: ribonucleic acid
RS: riboswitch
SBDD: structure-based drug design
TAR: transactivation response
TPP: thiamine pyrophosphate
VS: virtual screening
