# Supplementary material for: Comparative Evaluation of Explicit Solvent Models for RNA-Ligand Docking
Source: J Chem Inf Model. 2026 May 21;66(11):6719–32. doi: 10.1021/acs.jcim.6c00498 (PMC13250911; doi:10.1021/acs.jcim.6c00498)
Supplement: Supplementary file 1 [file ci6c00498_si_001.pdf]

Supporting Information:

Comparative Evaluation of Explicit Solvent Models for RNA-Ligand Docking

Laura Almena Rodriguez[1], *Christian Kersten*[1, 2] *

[1] Institute of Pharmaceutical and Biomedical Sciences, Johannes Gutenberg-University Mainz, Staudingerweg 5, 55128 Mainz, Germany.

[2] Institute for Quantitative and Computational Biosciences, Johannes Gutenberg-University, BioZentrum I, Hanns-Dieter-Hüsch Weg 15, 55128 Mainz, Germany.

*Corresponding author: kerstec@uni-mainz.de

Content

1. Additional Figures S1 – S5 .... S2
2. References S8

1. Additional Figures S1 – S5

FlexX and HYDE

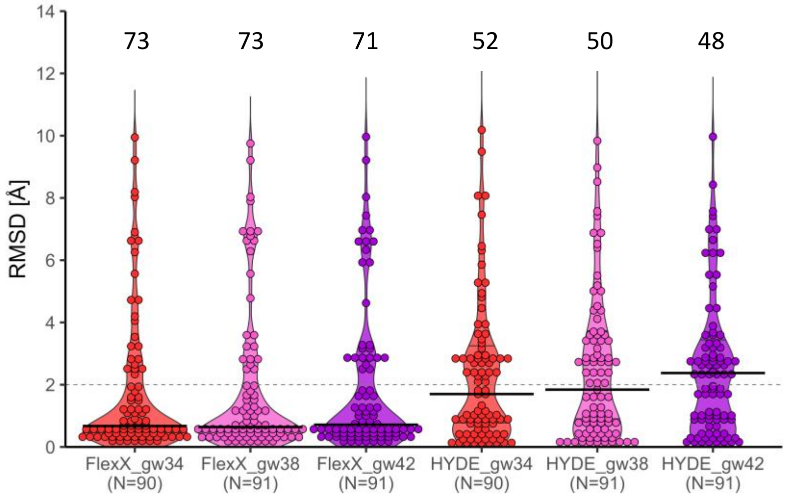

Figure S1: Redocking RMSD values across different Galaxywater-CNN (*gw*) score cut-offs summarized as violin plots for *gw* score cut-off ≥34 (red), ≥38 (pink) and ≥42 (purple). FlexX and HYDE were used as docking tools. Distribution centers and variability are indicated by median (bold line) as well as first and third quartiles (thin lines). An RMSD value of 2.0 Å (dotted line) is used as cutoff for successful redockings and success rates in % are given above the violines. Raw data is shown in Table S1.

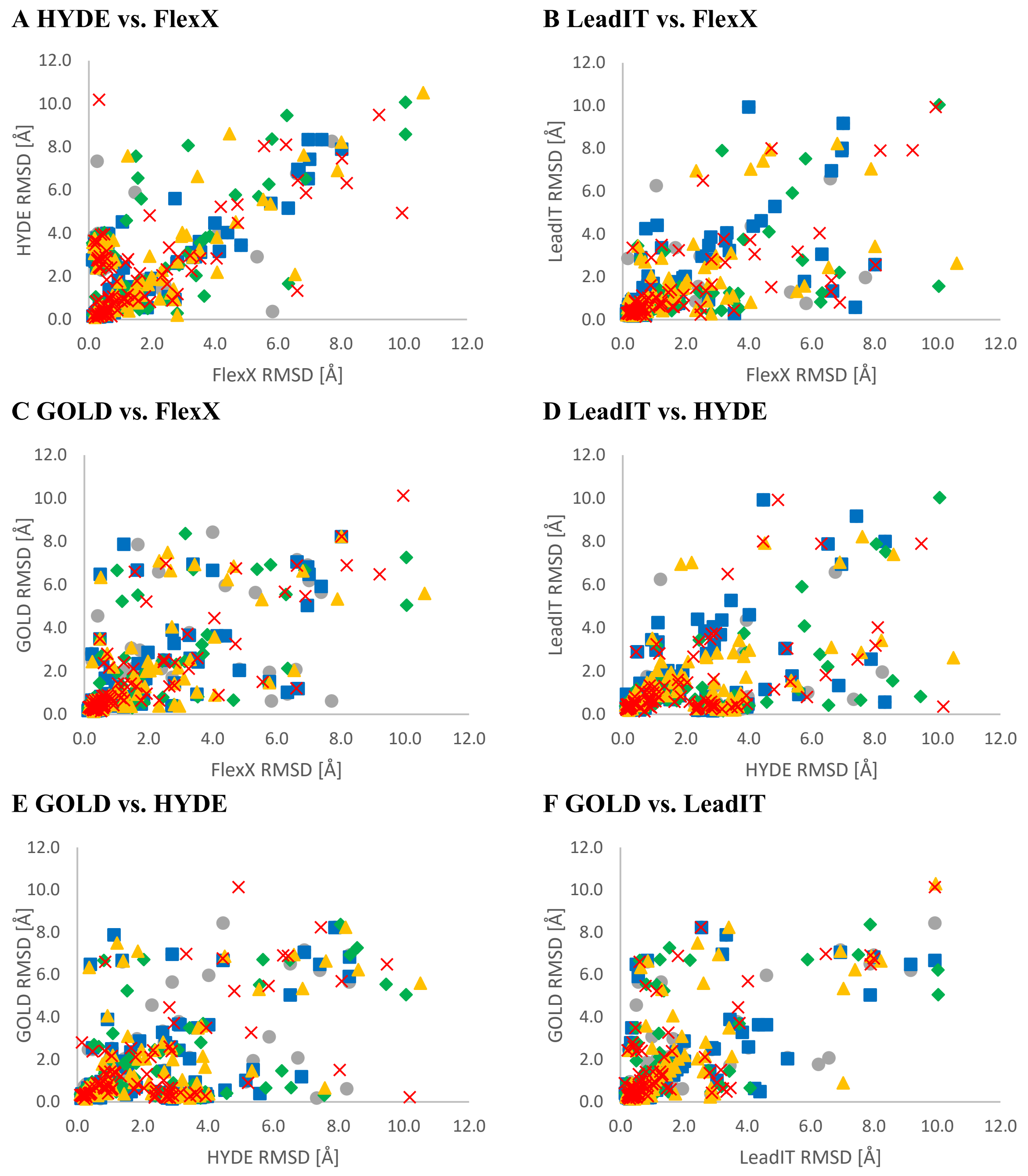

Figure S2. Scatter plots correlating redocking RMSD values in Å of two docking tools for: **A)** HYDE RMSD values plotted against FlexX RMSD values, **B)** LeadIT RMSD values plotted against FlexX RMSD values, **C)** GOLD RMSD values plotted against FlexX RMSD values, **D)** LeadIT RMSD values plotted against HYDE RMSD values, **E)** GOLD RMSD values plotted against HYDE RMSD values, **F)** GOLD RMSD values plotted against LeadIT RMSD values. Grey circles represent *dry* dockings, blue squares to *wet* dockings, green rhombus to *rism* dockings, yellow triangles to *waterdock_fxx* dockings and red crosses to *gw34* dockings.

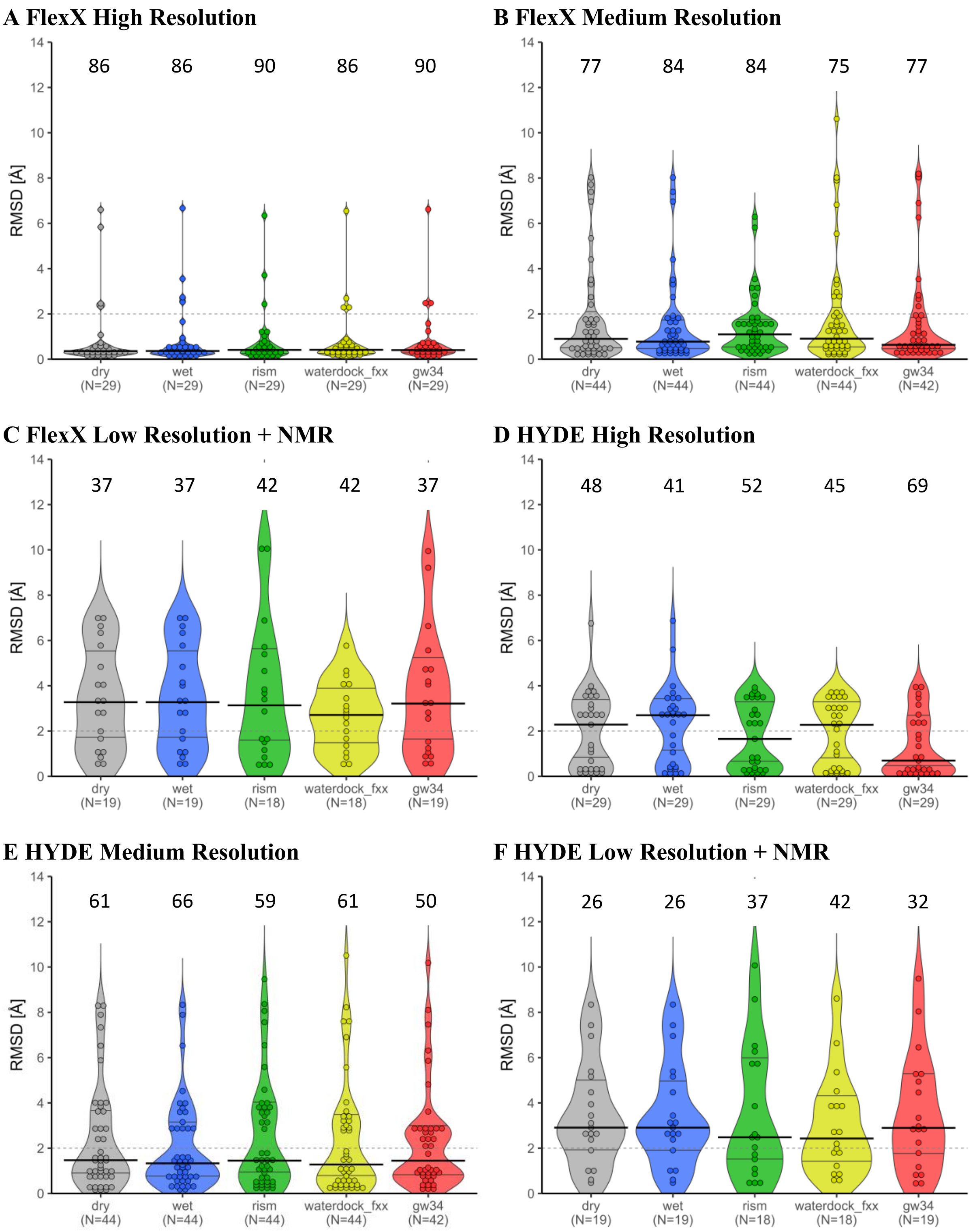

Figure S3: Redocking RMSD values across different solvent models summarized as violin plots for dry (grey), wet (blue), rism (green), waterdock_fxx (yellow) and gw34 (red) dockings. Results are grouped by high-resolution (≤2.0 Å) structures, medium-resolution (2.0–3.0 Å) structures, low-resolution (>3.0 Å) + NMR structures for docking tools: **A–C)** FlexX**, D–F)** HYDE, **G–I)** LeadIT, **J–L)** GOLD. Distribution centers and variability are indicated by median (bold line) as well as first and third quartiles (thin lines). An RMSD value of 2.0 Å (dotted line) is used as cutoff for successful redockings and success rates in % are given above the violines. Raw data shown in Table S1.

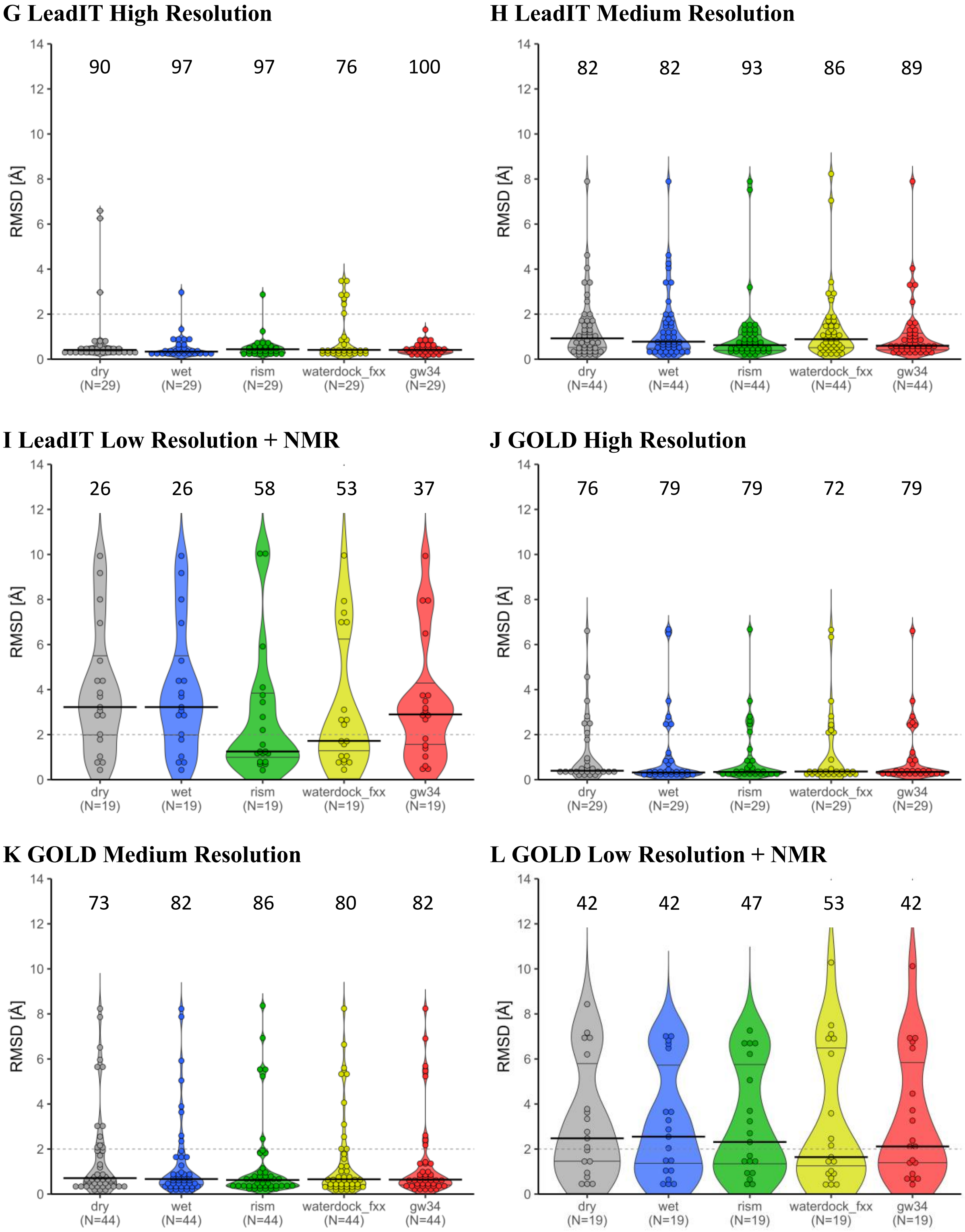

Figure S3 continued: Redocking RMSD values across different solvent models summarized as violin plots for dry (grey), wet (blue), rism (green), waterdock_fxx (yellow) and gw34 (red) dockings. Results are grouped by high-resolution (≤2.0 Å) structures, medium-resolution (2.0–3.0 Å) structures, low-resolution (>3.0 Å) + NMR structures for docking tools: **A–C)** FlexX**, D–F)** HYDE, **G–I)** LeadIT, **J–L)** GOLD. Distribution centers and variability are indicated by median (bold line) as well as first and third quartiles (thin lines). An RMSD value of 2.0 Å (dotted line) is used as cutoff for successful redockings and success rates in % are given above the violines. Raw data shown in Table S1.

FlexX

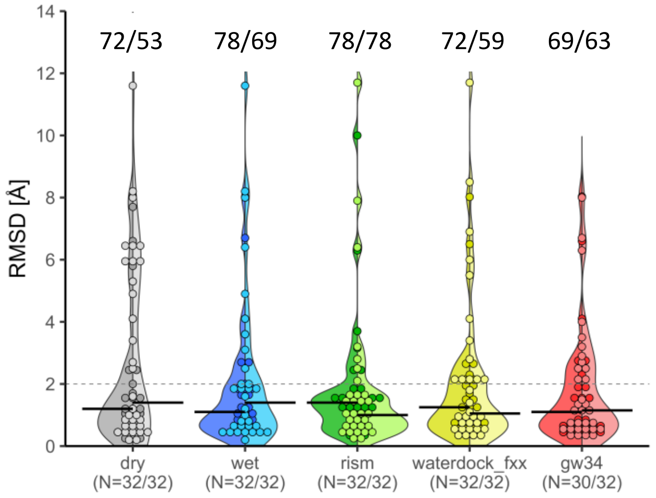

Figure S4: Redocking RMSD values across different solvent models summarized as split violin plots for dry (grey), wet (blue), rism (green), waterdock_fxx (yellow) and gw34 (red) FlexX dockings. Left half of the violine plot corresponds to dockings including ions and right half to ion-free dockings. An RMSD value of 2.0 Å (dotted line) is used as cutoff for successful redockings and success rates in % are given above the violines. Distribution centers and variability are indicated by median (bold lines). Raw data shown in Table S3.

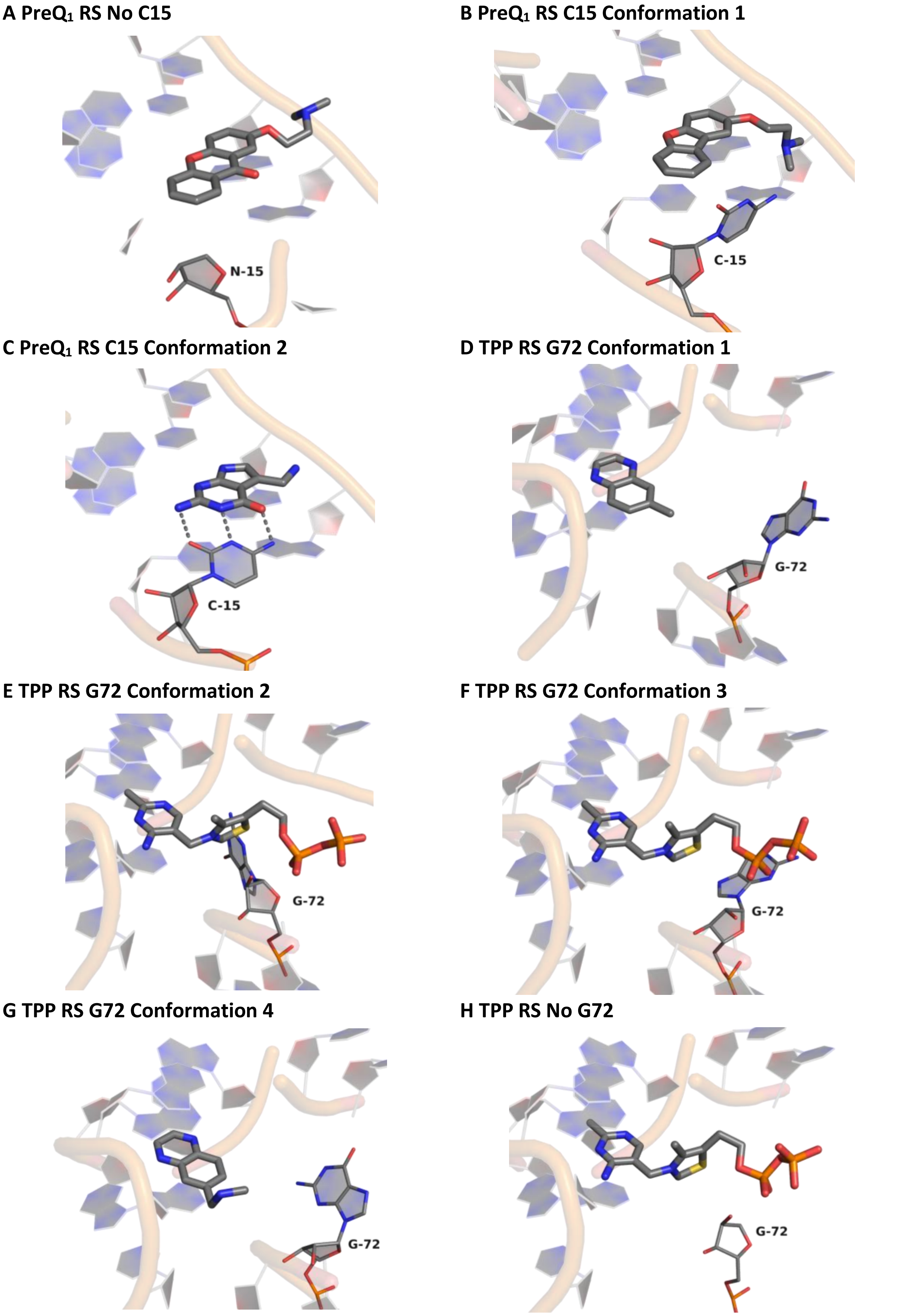

Figure S5: Representative ion- and solvent-free X-ray structures to visualize the different conformations of C15 for the PreQ$_1$ class I RS with **A**) no C15 (PDB 8YAM[1]), **B**) C15 in conformation 1 (PDB 6E1U[2]), **C**) C15 in conformation 2 (PDB 3Q50[3]) and G72 for the TPP RS with **D**) G72 in conformation 1 (PDB 7TZT[4]), **E**) G72 in conformation 2 (PDB 2GDI[5]), **F**) G72 in conformation 3 (PDB 7TDA[6]), **G**) G72 in conformation 4 (PDB 7TZR[4]) and **H**) no G72 (PDB 2HOK[7]). Dotted lines represent polar contacts.

2. References

(1) Parmar, S.; Bume, D. D.; Conelly, C.; Boer, R.; Prestwood, P. R.; Wang, Z.; Labuhn, H.; Sinnadurai, K.; Feri, A.; Ouellet, J.; Homan, P.; Numata, T.; Schneekloth, J. S. Mechanistic Analysis of Riboswitch Ligand Interactions Provides Insights into Pharmacological Control over Gene Expression. February 23, 2024. https://doi.org/10.1101/2024.02.23.581746.

(2) Connelly, C. M.; Numata, T.; Boer, R. E.; Moon, M. H.; Sinniah, R. S.; Barchi, J. J.; Ferré-D'Amaré, A. R.; Schneekloth, J. S. Synthetic Ligands for PreQ1 Riboswitches Provide Structural and Mechanistic Insights into Targeting RNA Tertiary Structure. *Nat. Commun.* **2019**, *10* (1), 1501. https://doi.org/10.1038/s41467-019-09493-3.

(3) Jenkins, J. L.; Krucinska, J.; McCarty, R. M.; Bandarian, V.; Wedekind, J. E. Comparison of a PreQ1 Riboswitch Aptamer in Metabolite-Bound and Free States with Implications for Gene Regulation. *Journal of Biological Chemistry* **2011**, *286* (28), 24626–24637. https://doi.org/10.1074/jbc.M111.230375.

(4) Zeller, M. J.; Favorov, O.; Li, K.; Nuthanakanti, A.; Hussein, D.; Michaud, A.; Lafontaine, D. A.; Busan, S.; Serganov, A.; Aubé, J.; Weeks, K. M. SHAPE-Enabled Fragment-Based Ligand Discovery for RNA. *Proceedings of the National Academy of Sciences* **2022**, *119* (20). https://doi.org/10.1073/pnas.2122660119.

(5) Serganov, A.; Polonskaia, A.; Phan, A. T.; Breaker, R. R.; Patel, D. J. Structural Basis for Gene Regulation by a Thiamine Pyrophosphate-Sensing Riboswitch. *Nature* **2006**, *441* (7097), 1167–1171. https://doi.org/10.1038/nature04740.

(6) Zeller, M. J.; Nuthanakanti, A.; Li, K.; Aubé, J.; Serganov, A.; Weeks, K. M. Subsite Ligand Recognition and Cooperativity in the TPP Riboswitch: Implications for Fragment-Linking in RNA Ligand Discovery. *ACS Chem. Biol.* **2022**, *17* (2), 438–448. https://doi.org/10.1021/acschembio.1c00880.

(7) Edwards, T. E.; Ferré-D'Amaré, A. R. Crystal Structures of the Thi-Box Riboswitch Bound to Thiamine Pyrophosphate Analogs Reveal Adaptive RNA-Small Molecule Recognition. *Structure* **2006**, *14* (9), 1459–1468. https://doi.org/10.1016/j.str.2006.07.008.
